# Broadband 120 MHz Impedance Quartz Crystal Microbalance (QCM) with Calibrated Resistance and Quantitative Dissipation for Biosensing Measurements at Higher Harmonic Frequencies

**DOI:** 10.3390/bios6020023

**Published:** 2016-05-25

**Authors:** Manuel Kasper, Lukas Traxler, Jasmina Salopek, Herwig Grabmayr, Andreas Ebner, Ferry Kienberger

**Affiliations:** 1 Keysight Technologies Austria GmbH, Keysight Labs Linz, Gruberstrasse 40, 4020 Linz, Austria; manuel.kasper@keysight.com; 2 Johannes Kepler University Linz, Biophysics Institute, Gruberstrasse 40, 4020 Linz, Austria; Lukas.Traxler@jku.at (L.T.); herwigreinhard.grabmayr@students.fh-wels.at (H.G.); Andreas.Ebner@jku.at (A.E.); 3 Faculty of Science, Department of Chemistry, Division of Physical Chemistry University of Zagreb, Horvatovac 102a, 10000 Zagreb, Croatia; jsalopek@chem.pmf.hr; 4 University of Applied Sciences Upper Austria, School of Engineering, Stelzhamerstrasse 23, 4600 Wels, Austria

**Keywords:** quartz crystal microbalance, impedance analysis, biosensing, dissipation, higher harmonics

## Abstract

We developed an impedance quartz crystal microbalance (QCM) approach with the ability to simultaneously record mass changes and calibrated energy dissipation with high sensitivity using an impedance analyzer. This impedance QCM measures frequency shifts and resistance changes of sensing quartz crystals very stable, accurately, and calibrated, thus yielding quantitative information on mass changes and dissipation. Resistance changes below 0.3 Ω were measured with corresponding dissipation values of 0.01 µU (micro dissipation units). The broadband impedance capabilities allow measurements between 20 Hz and 120 MHz including higher harmonic modes of up to 11th order for a 10 MHz fundamental resonance frequency quartz crystal. We demonstrate the adsorbed mass, calibrated resistance, and quantitative dissipation measurements on two biological systems including the high affinity based avidin-biotin interaction and nano-assemblies of polyelectrolyte layers. The binding affinity of a protein-antibody interaction was determined. The impedance QCM is a versatile and simple method for accurate and calibrated resistance and dissipation measurements with broadband measurement capabilities for higher harmonics measurements.

## 1. Introduction

Quartz crystal microbalance (QCM) is an acoustic sensing technique which uses the inverse piezoelectric effect for measuring different properties of surrounding media, such as mass adsorption or fluid viscosity. The mass changes per area (Δm/A) due to thin films deposited on the surface of a periodically-oscillating quartz crystal are related to a change in resonance frequency according to Sauerbrey’s equation [1], $\Delta f=-\frac{2\cdot n\cdot f_{0}^{2}}{\sqrt{\rho_{q}\cdot \mu_{q}}}\cdot \frac{\Delta m}{A}$, where Δf is the change in resonance frequency, $f_{0}$ is the fundamental resonance frequency and $\rho_{q}$ and $\mu_{q}$ are the density (2.947 × 10^11^ g·cm^−1^·s^2^) and shear modulus of quartz (2.648 g·cm^−3^), respectively. Due to its extraordinary mass sensitivity (<0.4 ng/cm^2^ for a 5 MHz quartz crystal), the technique was used originally for gravimetric measurements [2]. Due to the introduction of an empirical model to describe the influence of viscosity and density of liquids on the change in resonance frequency [3,4], the applications were extended including process control, environment engineering, as well as bio-medical diagnosis. Biochemical functionalization of the quartz crystal electrode allows a label-free detection of specific molecular interactions with high sensitivity and specificity and a real-time determination of kinetic rates and affinity constants. This makes QCM a versatile bioanalytic screening tool for various applications ranging from the detection of single molecular monolayers, polymer films, complex macromolecules, to whole cells [5,6,7]. While the QCM tracks changes in the resonant frequency due to different fluid properties or mass deposition on the quartz surface, it offers additional advantages over similar biosensing techniques such as surface plasmon resonance [8,9]. As additional mass on the quartz plate does not only affect its oscillation frequency but also changes the oscillation behavior (more specifically the motional resistance), these changes correlate with the energy loss per stored energy during one oscillation cycle (referred in the following as dissipation factor) and provide information about the mechanical properties of the deposited mass. Rigid materials which are coupled tightly to the quartz plate will not influence the motional resistance significantly as they follow the oscillations more or less directly. By contrast, elastic materials such as cells or lipid layers with entrapped water are not following the oscillations perfectly which leads to internal frictions and, therefore, energy dissipation. Thus, dissipation serves as an additional parameter characterizing the deposited mass layer. This additional information is critical for many biomolecular systems, including the investigation of conformational effects of ligand binding to a DNA complex [10], DNA hybridization, and DNA–protein interactions [11,12], or protein adsorption to biomaterials [13,14,15,16].

With respect to QCM measurement technology, various approaches exist to read out the resonance frequency of quartz crystals [17,18]. In the following we describe three approaches, including single frequency oscillators, time domain-based methods, and advanced impedance analysis. *Method 1*: In the single frequency oscillator method a harmonic oscillator is implemented around the quartz under test (QUT) involving an amplifying circuit and active amplitude control to maintain a pre-defined oscillation level. A second control loop acts on the phase lag and maintains a zero degree phase-shift between QUT current and voltage, as the QUT is an inherent part of the oscillator circuitry, its stability and noise performance directly affects the measurement result, and it is not easily possible to use advanced techniques, like calibration and averaging for improving the signal-to-noise ratio. Additionally, only a limited frequency band can be covered with this approach limiting broadband harmonic frequency operation. *Method 2*: Dissipation analysis is implemented in the time domain. The QUT is stimulated with short excitation bursts which have a frequency close to the relevant resonance. After the excitation is switched off, the QUT continues to oscillate at its natural resonance frequency with decaying amplitude due to energy dissipation [19]. This decaying oscillation is acquired in the time domain and the resulting data is processed in order to extract the resonance frequency and the dissipation coefficient. Similar to the impedance analysis method the excitation can be in a broad frequency range allowing harmonic operation up to, typically, 70 MHz. *Method 3*: In standard impedance analysis the QUT impedance is analyzed in a certain frequency range around the relevant resonance frequency. By sweeping the frequency, data from various frequencies is acquired and advanced averaging and curve fitting can be applied. An excitation voltage is put at the QUT terminals and both the voltage and the flowing current are measured while the impedance is calculated. There are several methods to implement this impedance analyzer approach [20], and in each of them the circuitry parts for signal generation and measurement are completely separated which allows to optimize each part individually, resulting in a better signal-to-noise ratio. Furthermore, sensitive input amplifiers and high quality connections enables low drive level operation thus reducing the mechanical stress and the power dissipation in the QUT.

In the following we present an advanced impedance based QCM workflow (*Method 3*) that works up to 120 MHz allowing for higher harmonics measurements as well as accurate calibration of resistance and dissipation. For an avidin-biotin test sample we compare the performance of the impedance QCM with the single frequency oscillator (*Method 1*) resulting in better signal-to-noise on the resistance. Metrological grade impedance calibration procedures are used to get calibration of the resistance and the dissipation with a sensitivity that is better compared to time domain based dissipation methods (*Method 2*). Additionally we show overtone higher harmonics measurements of up to 11th order using the impedance QCM.

## 2. Materials and Methods

### 2.1. Measurement Setup and Liquid Chamber

**Figure 1:**
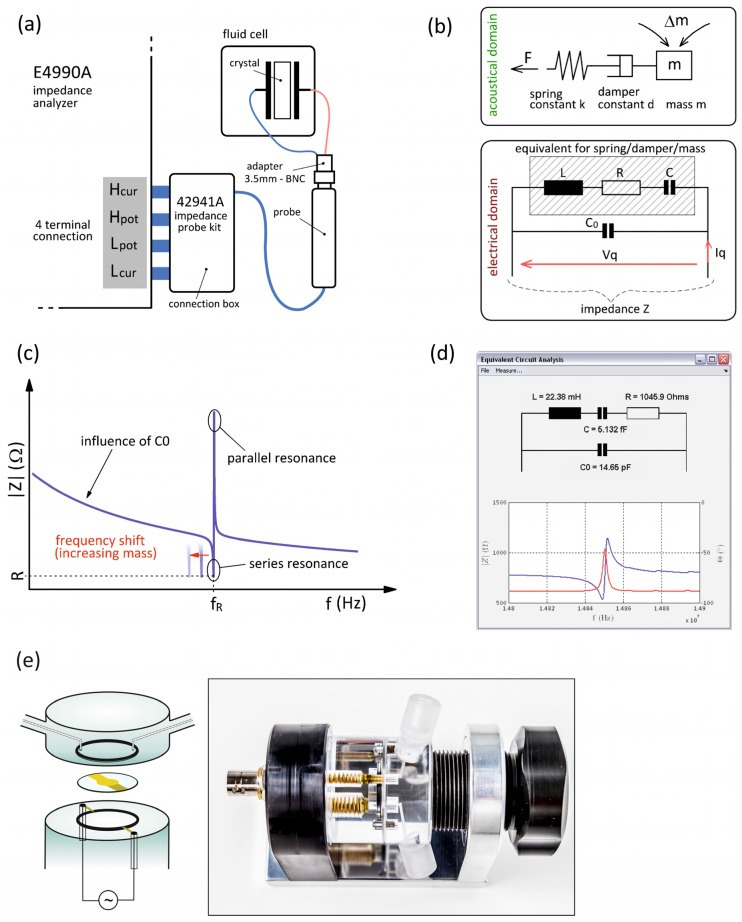
QCM setup and models. (**a**) Sketch of the impedance QCM setup. The impedance probe is used to connect the impedance analyzer with the liquid chamber; (**b**) mechanical (top) and electrical equivalent model (bottom) of the quartz resonator. V_q_ and I_q_ represent the voltage and the current applied to the quartz crystal, respectively. The impedance Z is equal to V_q_/I_q_; (**c**) sketch of a single resonance at f_R_ including the series and parallel resonance characteristics. For increasing mass adsorption the frequency is shifted to lower values (red arrow). The minimum impedance R represents the losses in the system. The electrode capacitance C_0_ determines the impedance curve at off-resonance frequencies; (**d**) graphical user interface showing the equivalent circuit LCR analysis based on the impedance analyzer built in software functions. The measured impedance resonance is shown including the absolute impedance in blue (Ω) and the phase in red (degree); and (**e**) Left: sketch of the liquid cell and quartz crystal with the upper part providing the fluidic channels and the lower part the electrical contacts to the quartz electrodes. Right: The impedance analyzer is connected to the assembled liquid cell via a BNC-connector at the liquid cell holder.

For all QCM measurements an in house developed liquid chamber (Johannes Kepler University Linz, Austria, http://www.jku.at/biophysics) consisting of two parts was used (Figure 1e). The lower part holds the quartz crystal and provides the electrical contacts, whereas the upper part contains the tube connections together with the inlet and outlet fluidic channels. By putting the two parts together, the quartz crystal gets clamped between the two x-rings. The liquid chamber is then mounted in the flow-cell holder and fixed by a screw. The electrodes are contacted by two corresponding counter-electrodes installed in the liquid cell. The wiring from these electrodes to the calibration plane where the actual impedance is measured is very short in order to avoid significant parasitic circuit elements, such as series inductance. For this reason, the electrical connection to the impedance analyzer is provided by a BNC socket directly at the liquid cell. To suck the liquid through the liquid chamber a constant liquid flow rate is provided by an automatic programmable syringe pump (NE-1000, New Era Pump Systems Inc., New York, NY, USA). In this study, the running buffer was either degased phosphate buffered saline (PBS, pH 7.3) or 0.5 M NaCl aqueous solution.

### 2.2. Biological Interaction Analysis and Polyelectrolyte Formation Measurements

For the avidin-biotin-IgG biochemical test measurement, AT-cut quartz crystals (ICM, Oklahoma City, OK, USA) with a diameter of 13.7 mm and a nominal fundamental resonance frequency of 9.995 MHz were used (surface area of working electrode was 20.47 mm^2^). At the beginning of the experiment, phosphate-buffered saline (PBS, 150 NaCl, 15 mM NaH_2_PO_4_, pH adjusted to 7.4) solution was run through the liquid chamber to equilibrate the system. As a first step of the biochemical reaction, NeutrAvidin, a deglycosylated version of native avidin (concentration 1.67 µM, molecular weight 60 kDa) known to adhere to bare gold surfaces [21], was injected into the liquid chamber via an automatic programmable syringe pump for approximately 15 minutes at a constant flow rate of 50 µL/min until saturation was reached. In saturation, a dense layer of NeutrAvidin is established on the gold electrode of the quartz crystal. Subsequent flushing with PBS solution was done in order to wash away loosely adsorbed NeutrAvidin. In the second step of the biochemical reaction, biotinylated goat IgG-antibody (concentration 0.5 µM, molecular weight 150 kDa, ~2 biotins per IgG) was injected into the liquid chamber for 15 min at a flow rate of 50 µL/min. In control measurements we observed an almost full saturation (>95%) of the biotin-binding sites of NeutrAvidin at a biotin-IgG concentration of 0.5 µM. Due to the high affinity between NeutrAvidin and biotin (K_d_ ≈ 10^−15^ M), biotin-IgG forms a stable complex with the NeutrAvidin layer. Again, after saturation the liquid chamber was flushed with PBS.

For the determination of the binding affinity of goat-IgG to protein G a similar approach was used. After adsorbing a chicken-avidin mutant (1.67 µM, described in detail in [22]) to the gold electrode of the quartz crystal, biotinylated goat IgG-antibody (0.5 µM) was bound on top of the avidin mutant layer. Upon saturation, bacterial cell wall protein G from *Streptococcus* sp. was injected at increasing concentrations ranging from 1.37 nM to 1000 nM (solutions prepared by a serial dilution by a factor of three) until reaching equilibrium. Before the next injection, the flow-cell was rinsed with PBS after each injection step.

For the formation of polyelectrolyte monolayers, a SiO_2_ coated 5 MHz quartz crystal was used (Q-Sense, Gothenburg, Sweden). Stock solutions of Poly(allylamine hydrochloride) (PAH, *M*_w_ = 15,000 g·mol^−1^, degree of amine functionalization *f* = 0.89) and Poly(sodium 4-styrenesulfonate) (PSS, *M*_w_ = 70,000 g·mol^−1^, degree of styrenesulfonate functionalization *f* = 0.83) were prepared by dissolving polyelectrolytes and NaCl in the aqueous solution of hydrochloric acid (*c*(HCl) = 0.1 mM) in order to assure the complete protonation of the polycation. Polyelectrolyte solutions were prepared of 50 mM (in monomer units) stock solutions by diluting the stock solutions with 0.1 mM·HCl. The concentration of the injected polyelectrolyte solutions was 1 mM (in 0.5 M NaCl). After exposing the sensor chip to the respective polyelectrolyte solution for approx. 10 min, it was rinsed with 0.5 M NaCl for approx. 5 min.

### 2.3. Impedance Analyzer, Impedance Probe, and Impedance Calibration

For impedance measurements the Keysight E4990 impedance analyzer (Keysight Technologies, Santa Rosa, CA, USA) is used within the frequency range of 20 Hz to 120 MHz. Alternatively, the Keysight 4294A impedance analyzer (Keysight Technologies) can also be used for frequencies of up to 110 MHz. Both impedance analyzers apply the auto-balancing-bridge measurement method [20] and have a four-terminal port for connecting the quartz crystal in the liquid chamber. The Keysight 42941A impedance probe (Keysight Technologies) is used to connect the impedance analyzer to the liquid chamber. This probe effectively converts the four terminal connection into a single coaxial one and adds only little additional noise and drift. The probe is equipped with a SMA connector; therefore, the connection to the liquid chamber is done using a SMA-BNC adaptor (*cf.* Figure 1a).

For the QCM calibration a two-step procedure is applied. In the first step, calibration is done with the impedance probe by running a procedure including phase correction, open, short, and load measurements. The calibration measurements are done over the full frequency span (20 Hz–120 MHz). Short and load calibration standards are included in the impedance probe kit, while open is not an explicit standard. The calibration effectively shifts the measurement plane directly to the SMA connector of the impedance probe, thereby eliminating any effects of the probe connection cable. For this first step it is typically enough to recalibrate after several months. In a second calibration step the parasitic effects of the liquid chamber and the quartz crystal including stray inductance and electrode capacitance C_0_ are removed. This is done by a built-in fixture compensation assuming an open circuit and ignoring resonances. Due to the strong dependency on the individual quartz crystals this procedure is typically done before every measurement. The absolute impedance accuracy of the calibrated system is well within 1% for fundamental measurements and 3% for overtone measurements and traceable to metrology standards. The frequency reference is provided by a temperature-controlled time base resulting in 1 ppm accuracy and 0.5 ppm drift over a one year period.

### 2.4. QCM Models and Data Analysis

Figure 1a shows the QCM setup and Figure 1b shows a lumped circuitry model for the QCM in the acoustic domain and in the electrical domain. In the acoustic domain the oscillation mode is a thickness shear wave modelled as a harmonic spring-mass-damper oscillator. Based on the piezoelectric effect the quartz crystal acts as transformer between the acoustical and the electrical domain, thereby converting forces and displacements into currents and voltages, respectively. By measuring the electrical impedance Z ($Z=\frac{V_{q}}{l_{q}}$) between the two connectors of the quartz, the mechanical elements are replaced by an electrical equivalent circuitry model. This leads to an L-C-R equivalent circuit which, is also called the motional leg. The LCR circuit is in parallel to a static shunt capacitance C_0_ which represents the electrode capacitance of the quartz crystal. This capacitance is a purely electric effect which has no equivalent in the acoustic domain. The complete electrical equivalent circuit is known as Butterworth–Van Dyke model. Figure 1c shows a sketch of a single resonance curve obtained from an LCR circuit. Two characteristic resonances, the series resonance and the parallel resonance, can be obtained. The first one is the serial resonance where the magnitude of the impedance drops to a minimum value R representing the losses in the system. The series resonance is characterized by the resonance frequency f_R_ and the quality factor Q, with the former given by $f_{R}=\frac{1}{2\pi \sqrt{L\cdot C}}$. The quality factor represents the losses of the system at resonance and is the ratio of stored energy and dissipated energy. Thus, it is influenced by both, the reactive components L and C, as well as the dissipative element R. The quality factor Q is given by $Q=2\pi \cdot f_{R}\frac{L}{R}$. The inverse of the quality factor is called dissipation coefficient d = 1/Q. Both quantities are dimensionless and without units. Typical *Q*-values for the used quartz crystals are in the range of 1000 to 3000 in liquid environment and up to 100,000 in air. The parallel electrode capacitance C_0_ causes the subsequent parallel resonance at high impedance where the motional leg becomes inductive at frequencies above the series resonance. The parallel resonance is however not used in QCM. At off-resonance frequencies the total impedance is defined mostly by the electrode capacitance C_0_. Figure 1d shows the quantitative equivalent circuit analysis that is included in the impedance analyzer. To determine the circuit element values L, C, R, and C_0_, a frequency sweep is performed around the resonance frequency with the C_0_ compensation switched off. For instance, to characterize a 10 MHz quartz crystal a sweep between 1 MHz and 15 MHz is applied.

### 2.5. QCM Software Control

A graphical user interface (GUI) that controls the E4990 impedance analyzer has been implemented in MATLAB. The GUI monitors the resonance frequency over time as well as the corresponding resistivity. The GUI allows to set up the measurement, start-and-stop the data record, and has a built-in conversion model to calculate the quantitative mass adsorption based on the frequency shift. Dissipation values are calculated based on the measured resistance values. Before the measurement is started a wide span frequency sweep is done in order to extract the required equivalent circuit parameters (L, C, R, and C_0_) using the impedance analyzer built-in circuit analysis software. For the typically high Q (quality factor) value resonances the effect of C_0_ is a slight offset of the absolute resonance frequency. Therefore, fixture compensation is applied eliminating the effect of C_0_ and parasitic circuit elements arising from the liquid cell. Subsequently, a search algorithm is used to find the relevant resonance frequencies. The implementation is done using the interpolation capabilities and corresponding tracking search markers of the impedance analyzer. The actual frequency sweep is software triggered every 0.5 s corresponding to a refresh rate of 2 Hz. If a frequency shift larger than 200 Hz occurs (e.g., due to air bubbles in the liquid cell) the frequency tracking could be lost. This case is detected and the resonance search is redone automatically.

## 3. Results and Discussion

### 3.1. Biological Interaction Analysis

**Figure 2:**
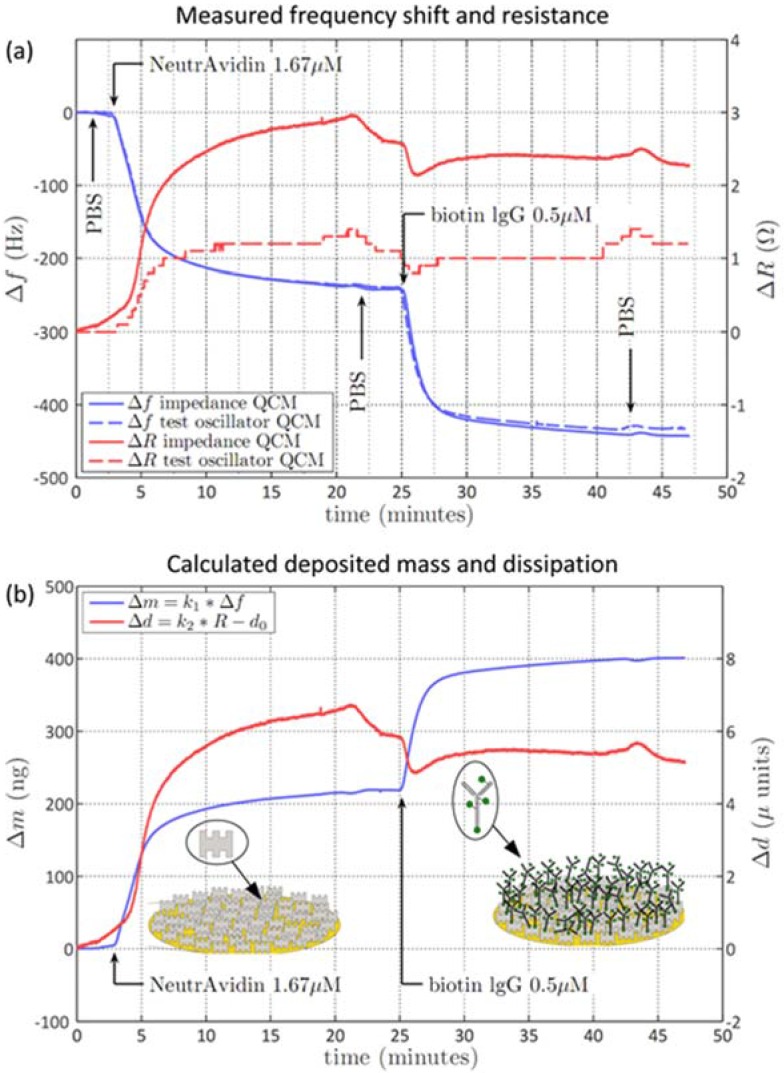
Impedance QCM measurement results of a simple bio-chemical test reaction. (**a**) The impedance QCM raw data (solid blue and red lines) is compared to the test oscillator QCM (dashed lines) (both with 9.995 MHz quartz crystals). Blue traces show frequency changes (Hz, left axis), and red traces show resistance changes (Ohm, right axis). After washing the liquid chamber with PBS (phosphate-buffered solution) the first step in this reaction is the adsorption of NeutrAvidin on gold (240 Hz shift) followed by the specific binding of biotin labeled antibodies (IgG, immunoglobulin G; 195 Hz shift); (**b**) based on the raw data the mass adsorption (ng; left axis) and the change in dissipation (micro-units; right axis) are calculated. The mass adsorption is inversely proportional to the frequency shift (k_1_), while the dissipation is proportional (k_2_) to the resistance. The sketches show the adsorption of avidin to the gold surface and the subsequent specific binding of biotin-IgG to the avidin layer.

For testing the performance of the calibrated impedance QCM we performed biosensing on the well characterized receptor-ligand system avidin-biotin which is known to be stable, robust, and having a high affinity [23]. Figure 2a shows the results of an avidin-biotin measurement on a 10 MHz fundamental frequency quartz crystal monitored with the impedance QCM (solid line) and compared to a commercial test oscillator QCM (dashed lines; Maxtek RQCM, Inficon). The first step of the biochemical reaction was to inject NeutrAvidin into the liquid cell leading to adsorption to the gold surface with a corresponding frequency shift of 240 Hz. In the second step biotinylated goat IgG-antibody was injected that binds specifically to the NeutrAvidin layer via biotin resulting in frequency shift of 195 Hz (*cf.* Materials and Methods). The frequency shift for both steps was the same either measured with the impedance QCM or the test-oscillator QCM. However, the corresponding resistance changes are significantly better resolved with the impedance QCM (Figure 2a, right axis), and a resistance change of 2.5 Ohms and 1 Ohm was obtained for the first and second step, respectively. The impedance-QCM results in a more than two times larger resistance signal compared to the test oscillator QCM. An accurate impedance calibration using open, short, and load measurements was done prior to the QCM experiments (*cf.* Materials and Methods). For the impedance QCM the absolute impedance accuracy of the calibrated system is better than 1% for fundamental resonance frequencies. Relative impedance measurements can be even done at higher sensitivity, for instance a 50 mΩ change can be easily detected on top of a 1 kΩ signal. Similarly, using the impedance QCM the absolute frequency is measured with 1 ppm accuracy while the sensitivity for frequency changes is below 0.05 ppm. As such, the highly accurate measurement capabilities of the impedance analyzer result in stable QCM frequency and resistance measurements. Additionally, due to the open, short, and load calibration routine using the impedance probe kit, the resistance values are also calibrated and traceable to metrology standards.

Figure 2b shows the mass adsorption (left axis) and the dissipation changes (right axis) calculated from the raw frequency and resistance data. The mass uptake was calculated using Sauerbrey’s equation which provides an inverse relationship to the frequency shift, given by $\Delta m=-\frac{A\cdot \sqrt{\rho_{q}\cdot \mu_{q}}}{2\cdot n\cdot f_{0}{}^{2}}\cdot \Delta f\;=-9.05\cdot 10^{-10}\;\cdot \;\Delta f$ (*cf*. Introduction). The first step of NeutrAvidin adsorption to the gold surface results in a mass uptake of 218 ng. Based on the NeutrAvidin molecular weight of 60 kDa this corresponds to 2.19 × 10^12^ NeutrAvidin molecules bound to the gold electrode, which is in good accordance to other studies [21]. The second step of biotin-IgG binding results in a mass uptake of 177 ng, corresponding to 7.11 × 10^11^ biotin-IgG molecules (molecular weight of 150 kDa). The factor of three lower number of biotin-IgG molecules, compared to NeutrAvidin, corresponds well to the theoretical number of biotin-IgG molecules that are bound to the NeutrAvidin monolayer, assuming a size of the antibody based on its 3D structure [24]. The red trace in Figure 2b represents the change in the dissipation calculated from the resistance data and the model parameters of the quartz equivalent circuit for the corresponding frequency (L, C, R, C_0_). The equivalent circuit parameters were measured by acquiring an impedance sweep of sufficient bandwidth (*cf.* Materials and Methods). Based on the impedance sweep the equivalent circuit analysis software from the impedance analyzer automatically estimates the circuit parameters L, C, R, and C_0_ (*cf.* Figure 1d). The dissipation (d) is proportional to the resistance according to d=R/(ω × L), with R being the measured resistance, L the equivalent circuit inductance value and ω the circular frequency (ω = 2πf). A change in dissipation of 6.5 µU (micro units) and 5.5 µU was obtained for the NeutrAvidin adsorption and the biotin-IgG binding, respectively. The dissipation values are mainly related to the layer viscoelasticity and layer softness and describe structural changes within the deposited mass. The dissipation values were compared to commercially available time domain-based dissipative QCM and similar values were obtained on the same biochemical reaction (data not shown). Based on the accurate resistance measurements and equivalent circuit parameters determination, very small dissipation coefficients below 0.05 µU can be detected without special efforts with the impedance QCM. Using averaging and curve fitting methods a minimum detection level of 0.01 µU is possible with the impedance analyzer which is better than time domain based dissipation methods that typically have a minimum detection level of 0.03 µU.

**Figure 3:**
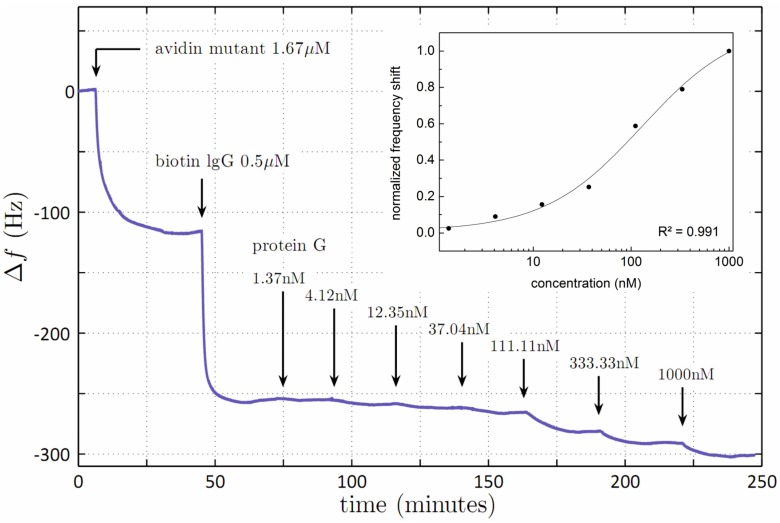
Impedance QCM measurement for the determination of the binding affinity of goat-IgG to protein G. Upon immobilization of biotinylated goat-IgG on a gold-adsorbed avidin-mutant layer, protein G was injected at different concentrations ranging from 1.37 nM to 1000 nM. For each step, the flow-cell was flushed with PBS after reaching equilibrium. Inset: Semi-logarithmic plot of normalized frequency shift per injection *versus* protein G-concentration, fitted with a logistic function.

In Figure 3 we show the application of the impedance QCM for the quantitative determination of the binding affinity of goat-IgG to protein G based on the above described immobilization of biotinylated goat-IgG on top of a gold-adhered avidin mutant layer. Protein G, at seven different concentrations ranging from 1.37 nM to 1000 nM, was injected until reaching equilibrium. The respective decrease in resonance frequency per injection step was normalized, plotted against protein G concentration, and fitted with a logistic function (Figure 3 inset), yielding an equilibrium dissociation constant K_D_ of 129 ± 38 nM.

### 3.2. Formation of Polyelectrolyte Multilayers

**Figure 4:**
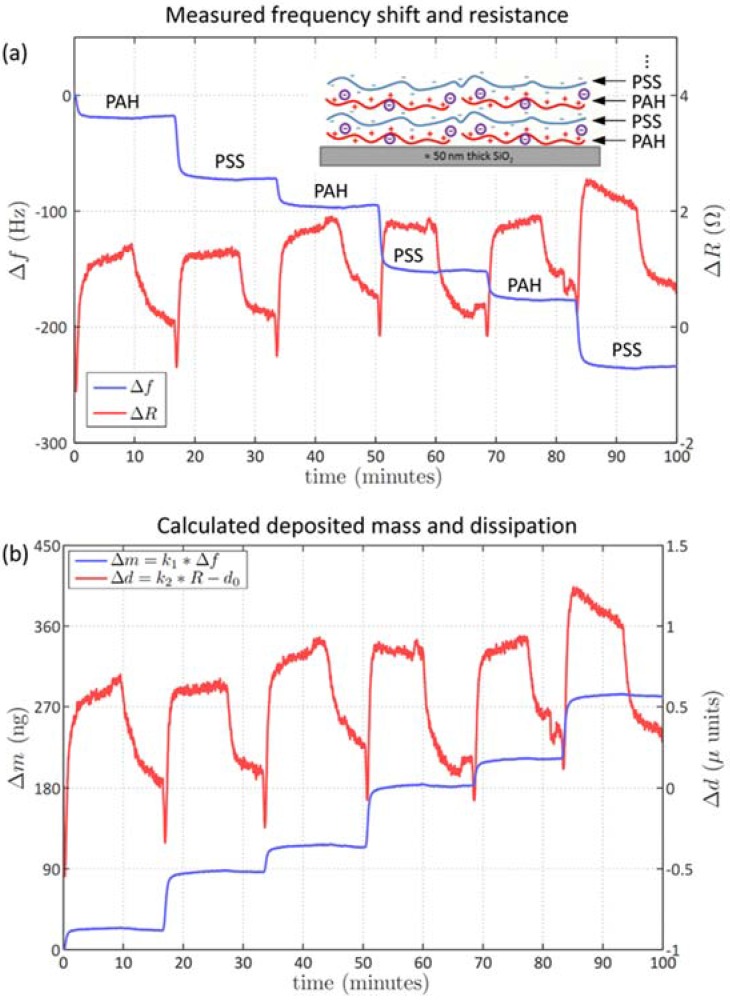
Impedance QCM measurement results of the formation of a polyelectrolyte multilayer (PEM) on top of a silicon dioxide coated quartz crystal (5 MHz resonance frequency). (**a**) Experimental data showing the stepwise decrease in resonance frequency (left axis, third harmonic, not normalized) and resistance (right axis) due to the sequential injection of solutions of poly(allylammonium hydrochloride) (PAH, 1 mM) and poly(sodium styrene sulfonate) (PSS, 1 mM) in the presence of NaCl (0.5 M). A 10 point moving average filter was applied to the raw data. The polymer was injected for 10 min at a flow rate of 50 µL/min followed by a 5 min injection of 0.5 M NaCl solution; and (**b**) calculated mass uptake (left axis) per electrode area and dissipation values (right axis) for the individual polymer layers.

For investigating multi-layer formation with the impedance QCM we studied the resonance frequency and resistive dissipation of a polyelectrolyte multilayer (PEM) on a silicon dioxide (SiO_2_)-coated quartz crystal (Figure 4). For the PEM formation, solutions of poly(allylammonium hydrochloride) (PAH) and poly(sodium styrene sulfonate) (PSS) were injected subsequently into the liquid chamber in a multi-step process (Figure 4a). Each individual injection led to a mass deposition of the respective polymer on SiO_2_ as indicated by the stepwise decrease in resonance frequency (Figure 4a, left axis). The corresponding dissipative changes of layer formation and trapping of water molecules are shown in the stepwise resistance data (Figure 4a, right axis). Figure 3b shows the calculated mass uptake per electrode area according to the Sauerbrey equation yielding, on average, 61 ng for PSS and 29 ng for PAH. One of the reasons for the larger mass adsorption of PSS compared to PAH is the higher mass per charge and monomer unit. The corresponding increase in dissipation during the multi-layer formation was 0.5 µU per polymer layer, followed by a decrease to the initial value during the rinsing with 0.5 M solution of NaCl. This indicates that the additional polymer layers do not have any significant viscoelastic impact and the layers behave like a homogenous rigid mass. However, due to the high sensitivity of our impedance based QCM, we were able to detect the difference in energy dissipation between tightly-assembled PEM layers and loosely-bound polyelectrolytes. Further QCM studies on the PEM will investigate layer thickness and nano-assembly with respect to different ionic strengths. It is expected that at higher ionic strengths the film thickness depends on the type of anion counterbalancing the polycation charge.

### 3.3. Higher Harmonic Impedance Measurements

**Figure 5:**
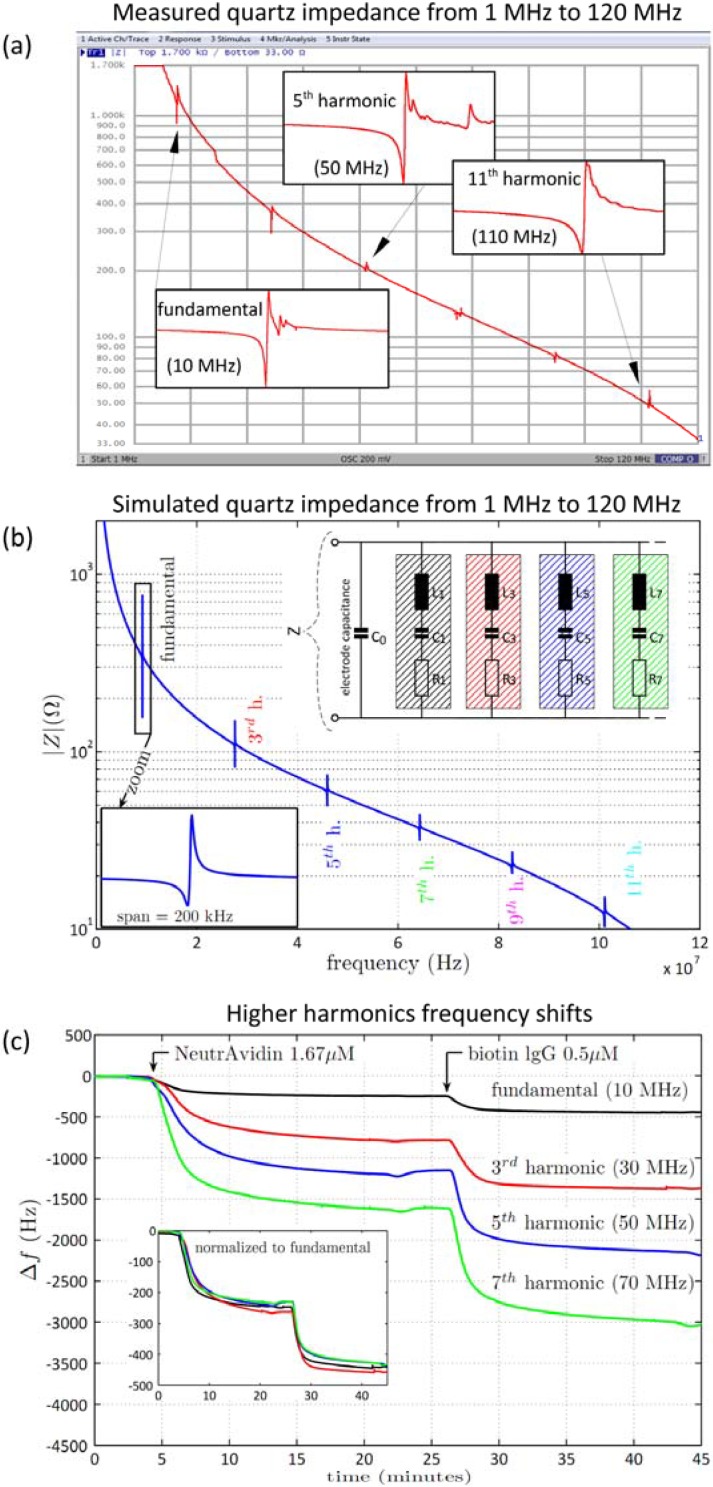
Harmonic measurements with the impedance QCM up to 120 MHz. (**a**) A broadband impedance sweep is done between 1 MHz and 120 MHz (1601 points). Based on the fundamental resonance frequency at 10 MHz, subsequent odd harmonic resonances are present up to 110 MHz corresponding to the 11th harmonics; (**b**) modeled impedance sweep using harmonic resonators. The individual resonances correspond to the multiple LCR circuits (sketches of LCR_1_, LCR_3_, LCR_5_, and LCR_7_ as inset) with the overall slope of the impedance curve mainly defined by the electrode capacitance C_0_; and (**c**) experimental results of the higher harmonics impedance QCM. The two step bio-chemical reaction (first step is NeutrAvidin adsorption to gold; second step is biotin-IgG binding to the avidin layer) is measured at the fundamental frequency and at higher harmonics (third, fifth, and seventh harmonics). For the harmonic frequencies, both the absolute frequency and the frequency shifts are multiple integer values of the corresponding fundamental values. The normalized frequency shifts are shown in the inset.

The impedance analyzer operates between 20 Hz and 120 MHz. Thus, higher harmonic resonances of the 10 MHz quartz crystal can also be investigated. Higher harmonics measurements have the advantage that the cheap, relatively thick, and easily-mountable 10 MHz crystal can be used at higher operating frequencies and it is not necessary to use a thinner and more expensive high-frequency quartz crystal. Figure 5a shows the measured broadband impedance spectrum of a pristine 10 MHz quartz crystal between 1 MHz and 120 MHz. The fundamental resonance at 10 MHz and the odd harmonics can be clearly identified up to the 11th harmonic at 110 MHz. For all quartz crystals and QCM setups, the even harmonic resonance numbers are not excited electrically. The overall impedance baseline falls with increasing frequency, which is caused by the parallel electrode capacitance C_0_. The electrode capacitance C_0_ is leading to a parallel resonance with a local impedance maximum that is following every series resonance with a local impedance minimum, resulting in the typical derivative resonance shape (*cf*. Figure 1). This resonance behavior can be obtained for all resonances and is shown as inset for the fundamental frequency, the 5th harmonics, and the 11th harmonics. The overall shape of the individual resonance curves is very similar. However, it can be observed that the quality factor decreases slightly at higher frequencies indicating increased losses. Additionally, spurious responses can be occasionally observed caused by additional resonant modes of the quartz crystal. Figure 5b shows a simulated impedance sweep in the same frequency range. The underlying circuitry model is shown in the inset and is composed of eleven parallel series resonant networks (only four LCR networks are shown in the sketch). A parallel shunt capacitance accounts thereby for the electrode capacitance C_0_, and the resonant frequency is related to the inductance (L) and capacitance (C). It is required that the L and C values fulfil the relationship L_n_ = L_1_/n and C_n_ = C_1_/n for each harmonic number n, with L_n_ and C_n_ being the values for the harmonic resonator inductance and capacitance, and C_1_ and L_1_ being the corresponding values for the fundamental resonance. The applied circuitry model matches the experimental broadband impedance sweep very well for both the fundamental frequency and higher harmonic resonances.

Figure 5c shows higher harmonics measurements of the biochemical two-step reaction of NeutrAvidin adsorption to gold and subsequent biotin-IgG binding to avidin. The frequency shift is shown for the fundamental frequency (~10 MHz), the third harmonic (~30 MHz), the fifth harmonic (~50 MHz), and the seventh harmonic (~70 MHz). Both steps of NeutrAvidin adsorption and biotin-IgG binding can be properly seen in all resonances at the same center frequency. The absolute frequency change thereby increases with the resonance number and the highest frequency change is obtained at the highest harmonic resonance number. This is in line with the model where the frequency change is related to the fundamental frequency f_0_ of the quartz crystal multiplied by the harmonic number n. Therefore, the expected frequency shift at higher harmonic oscillations is directly proportional to its order. Dividing each frequency shift by the harmonic number should, therefore, result in the same shift as measured at the fundamental resonance frequency. The inset of Figure 5c shows the normalized frequency shifts (Δf/n) for the harmonic and the fundamental frequency resonances. The curves are overlapping and the measured values are in good accordance to the expected behavior. This indicates that the assumptions of the Sauerbrey equation are fulfilled, including a thin, rigid, and uniformly distributed layer of NeutrAvidin and biotin-IgG. In addition to the 10 MHz quartz crystal we used a 25.4 mm diameter 5 MHz quartz crystal (Inficon, Inc., New York, NY, USA) and monitored the resonance frequency at the 23rd harmonic oscillation at 115 MHz over several hours in air (data not shown). For both quartz crystals the corresponding frequency curves are stable for several hours with a typical drift below 1 Hz/h, indicating the stability and high performance of the impedance analysis measurement.

## 4. Conclusions

An impedance QCM was used to measure the frequency shift and resistance for the multi-layer formation of polyelectrolytes and for specific avidin-biotin interaction. Furthermore, we applied this technique to determine and quantify the binding affinity between *Streptococcal* protein G and surface-bound goat-IgG. The broadband impedance measurement capabilities allow measurements between 20 Hz and 120 MHz including higher harmonic modes of up to 11th order for the 10 MHz fundamental resonance frequency quartz crystal. The Sauerbrey equation is used to convert the frequency shift into mass adsorption, while the dissipation is calculated from the measured resistance values. A full impedance calibration resulted in stable, quantitative and accurate resistance measurements with high sensitivity that is traceable to metrology standards. The calibration concept applied in this paper relies on traceable calibration standards (Short, Open, Load) which are consecutively connected to the impedance analyzer. By measuring the calibration standards first, all systematic errors due to different liquid cells, different cables, thermal drift and electrical connector degradation are corrected. This results in a significant accuracy improvement if small changes in impedance, respectively resistance and dissipation, are measured. We show this by measuring small resistance changes (0.3 Ω) at a high signal-to-noise ratio. The impedance calibrated QCM results in superior resistance signals compared to un-calibrated QCM systems, with respect to both the absolute accuracy and the relative signal-to-noise ratio. In un-calibrated systems where scale errors and offset errors are added to the real value of the physical resistance, the measurement of low absolute resistance values is not accurate. For instance, for the avidin adsorption on gold we measured a calibrated 2.5 Ω resistance change *versus* 1 Ω measured with an un-calibrated system. This corresponds to a relative error of 60% for un-calibrated systems. Comparing the performance of the method presented in this paper with traditional QCM methods, we can resolve smaller changes more robustly due to the advanced calibration and impedance measurement system. Additionally, quantitative measurements can be performed, and absolute dissipation values are comparable among different QCM machines. In summary, a high-performing impedance analyzer allows for fast and sensitive measurements in a broad frequency range, with the main advantage that calibrated and accurate resistance data can be acquired, which is important for robust dissipation analysis at higher modes. Future applications will focus on bio-medical detection of single virus particles in complex liquid environments where sensitivity, robustness and quantitative evaluation are of utmost importance.
